# Modern Sociology

**Published:** 1899-01-21

**Authors:** 


### Modern Sociology.

### THE RACIAL RELATIONS OF THE TRAMP.

Upon no one, except the criminal, has the condemnation of the sociologist or the penologist rested more heavily than upon the tramp. He is regarded by many as a mongrel between the criminal and the pauper, possessing the predatory instincts of the one, combined with the parasitic habits of the other. His ranks are the chief recruiting grounds for criminals, if not an actual school of crime, while his rapacity and indolence are a burden upon the thrift and a menace to the industry of the community. His large and increasing numbers are accordingly regarded as a threatening cloud above our social future, while from the singular constancy in successive generations, both of his physical type and of his relative numbers in the community, he has come to be regarded as a distinct, self-perpetuating sub-species of the *genus homo*.

While there is truth in all these views, except the last, yet we are convinced that a more careful and closer study of the class itself reveals that its dangerousness, both individually and racially, has been seriously over-estimated. So far as the immediate or individual injuriousness of the tramp is concerned, we shall content ourselves with saying that the "hobo," as he terms himself, is for the most part a weak, vain, harmless sort of a creature, slack of muscle and irresolute of mind—an arrant coward, dangerous only to the hen-roost and the clothes-line.

The "tax" which he levies upon society consists chiefly of broken victuals, cast-off clothing, stale beer, even absolute garbage. The only members of the community with whom he "competes" in the struggle for existence are the household watchdog and the family pig. His racial relations, however, are both peculiar and interesting, and such as, in our judgment, to almost deprive him of any injurious influence upon the future or the race-streams.

First of all he is obviously, by the very nature of his "profession," a man without a family. The idea of even supporting himself is abhorrent to him, let alone a wife and children. In behalf of his physical comfort he has renounced the family even as did the monk in behalf of his spiritual. And in response Nature has emphatically renounced him. There is no mate for the tramp, no female of his species. Matrimony he eschews on principle like certain other gentlemen of leisure, and the entire feminine sex despises and eschews him. Even the lowest and most ignorant class of women in the country districts which he infests during the summer look down upon the tramp with a measureless contempt and dislike, while in his winter quarters in the streets and slums his only female associates are the lowest class of prostitutes and criminals, who are almost absolutely sterile. There are very few women tramps, partly on account of the hardships incident to the life, but mainly because the "live-well-without-working" element which among men turns tramp, among women turns prostitute. No need for the female vagabond class to go on the road, the street is far enough. And, as has been shown for the prostitute by several investigations, neither class leaves many descendants, for which the community has reason to be thankful. But, it will be urged at once, if the tramp has no legitimate and very few illegitimate offspring, how are his ranks kept so well filled and his type so uniform? His ranks have an unfailing source of supply, for they are recruited from the failures of every class in the community, from the highest to the lowest. The sediment of every generation sinks naturally into them, by mere force of gravity. So long as society has failures it will have tramps. The uniformity of type is striking enough to tempt us to dub him a true species. There is no nationality among tramps, so far as physiognomy goes. The type can be recognised at sight in every country under heaven. A German tramp is almost the exact *replica* of an Irish one, and either of them far more like the other than he is to the remainder of his own countrymen. But this, again, does not imply continuity of descent; merely that the charms of a wandering and workless life appeal to much the same type of mind and body in each generation and country. The attraction is constant and unvarying, the response almost equally so. And as this type of temperament is that of the simple, nomadic savage, who slumbers more or less deeply in all of us, what wonder that even its physique is so uniform. Any individual peculiarities possessed by the recruit are quickly swamped by the uniform and discipline of the corps. Even his name disappears and is replaced by some descriptive soubriquet, Indian fashion, usually uncomplimentary, such as "Rags," "Ginger," or "Tubby."

The tramp is a drop in the side branch of the race-stream which flows out into the desert, never again to rejoin the parent river. In the language of the Weissman school he is all somato-plasm, the germ-plasm has died out of him. As the prostitute by the abuse, so he, by the rejection of the race-continuing instinct, has stamped himself as unfit to survive—fit for elimination. The wheat is gone, the remainder is "but chaff which the wind driveth away."

